# Prophylactic Antiarrhythmic Effect of Anesthetics at Subanesthetic Concentration on Epinephrine-Induced Arrhythmias in Rats after Brain Death

**DOI:** 10.1155/2015/575474

**Published:** 2015-01-14

**Authors:** Yuka Miyata, Mitsuo Iwasaki, Hiroo Yamanaka, Masanori Sato, Takahiko Kamibayashi, Yuji Fujino, Yukio Hayashi

**Affiliations:** ^1^Department of Anesthesiology, Osaka University Faculty of Medicine, Suita, Osaka 565-0871, Japan; ^2^Department of Anesthesiology, Sakurabashi-Watanabe Hospital, 2-4-32 Umeda, Kita-ku, Osaka 530-0001, Japan

## Abstract

The present study using brain death model of rats was designed to examine whether prophylactic administration of volatile anesthetics and propofol prevent the epinephrine-induced arrhythmias. A Fogarty catheter was placed intracranially for induction of brain death. After brain death, the rats were randomly assigned to five groups: the control group (no anesthetics), the sevoflurane group (0.8%), the isoflurane group (0.5%), the halothane group (0.3%), and the propofol group (195 *μ*g*·*kg^−1^ *·*min^−1^). These anesthetics were about 30% of ED_50_ of each anesthetic. The arrhythmogenic dose of epinephrine was determined in each anesthetic group. In addition, we examined left ventricular levels of connexin 43 phosphorylation 30 min after administration of each anesthetic with Western blot analysis. The arrhythmogenic dose of epinephrine in the sevoflurane group was significantly higher than that in the control group, while the arrhythmogenic dose of epinephrine in any other anesthetic group was not different. On the other hand, the ratio of phosphorylated-connexin 43/total connexin 43 was also similar among the study groups. Thus, prophylactic administration of subanesthetic dose of sevoflurane is effective in preventing epinephrine-induced arrhythmias after brain death, but phosphorylation of connexin is not involved in the antiarrhythmic property of sevoflurane.

## 1. Introduction

Brain death is often associated with significant physiological instability and inappropriate managing of potential donors may result in deterioration in organ functions before retrieval [1]. Arrhythmias are one of the common physiological incidents, presumably induced by myocardial damage following the catecholamine storm at the onset of brain death and control of the lethal arrhythmias, such as ventricular tachycardia and ventricular fibrillation, is critical for optimal perfusion to potential organs for transplantation [1].

Volatile anesthetics, including halothane, isoflurane, and sevoflurane, are known to inhibit several types of arrhythmias such as reperfusion arrhythmias [2]. In addition, propofol, a popular and short-acting intravenous anesthetic, was reported to suppress ischemic-induced ventricular arrhythmias [3]. Thus, prophylactic administration of these anesthetics may prevent the lethal arrhythmias after brain death. However, administration of anesthetics would have a potential risk of deteriorating cardiovascular variable, since myocardial function was depressed after brain death [1, 4]. Thus, it may be important whether these anesthetics can exert antiarrhythmic effect without aggravation of hemodynamic variables.

Therefore, the present study using brain death model of rats was designed to examine whether these anesthetic agents prevent the epinephrine-induced arrhythmias at subanesthetic concentration with little cardiovascular collapse. In addition, we investigated whether these anesthetics affect phosphorylation of connexin 43, which was known to be a principal cardiac gap-junction channel protein and involved in the genesis of ventricular arrhythmias [5].

## 2. Materials and Methods

The investigation conforms to the Guide for the Care and Use of Laboratory Animals published by the US National Institutes of Health (NIH publication number 82-23, revised 1996) and the study protocol was also approved by the Animal Care Committee of Osaka University Faculty of Medicine.

**Figure 1:**
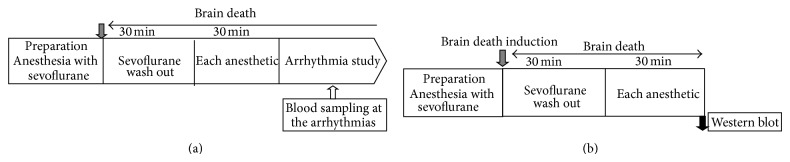
Diagram of the study protocol. (a) Determination of arrhythmogenic dose of epinephrine in each anesthetic group. (b) Western blot analysis.

Male Sprague-Dawley rats, weighing 350–430 g, were used and housed in a temperature controlled environment under 12-hour ligfht: 12-hour dark cycles, with free access to food and water. The rats were primarily anesthetized with 3.0% sevoflurane in oxygen. After tracheotomy, the lungs were mechanically ventilated with the tidal volume of 12 mL*·*kg^−1^ at 40–50 bpm (Rodent Ventilator; Ugo Basile, Varese, Italy). The ventilation rates were adjusted to maintain PaCO_2_ at 35–45 mmHg. Lead II of the electrocardiogram and heart rate was monitored continuously by ECG amplifier and pulse counter unit (AC-611G; Nihon Kohden, Tokyo, Japan). Heparin-filled polyethylene catheters (PE-50 and PE-10) were inserted into the carotid artery for blood sampling and pressure monitoring with a pressure transducer unit (AP-641G; Nihon Kohden) and into the jugular vein for administration of drugs. A heating pad was used to maintain rectal temperature at 37.5–38.5°C. Arterial pH and oxygen tension were maintained at 7.35–7.45 and more than 100 mmHg, respectively. Intravenous volume loading was carried out with Ringer's solution at a rate of 1 mL*·*kg^−1^ *·*min^−1^. Anesthesia was maintained for a further 30 min to achieve a steady state after completion of the preparation; subsequently, mean arterial pressure and heart rate were recorded as baseline data. Next, we inserted a 4 Fr Fogarty catheter into the animal's subdural space through a left frontoparietal burr hole. As reported previously [6], brain death was produced by graded inflation of the catheter by 300 *μ*L of saline, according to Pratschke et al. [7]. The development of complete electrocerebral inactivity, characterized by flat line of electroencephalography, apnea, areflexia, and maximally dilated and fixed pupils, was confirmed. After induction of brain death, we discontinued sevoflurane and held animals to wash out sevoflurane for 30 min, and then we recorded mean arterial pressure and heart rate. The study design is illustrated in Figure 1.

The animals (*n* = 42) were randomly assigned to the following 5 groups: the control group (*n* = 8), the sevoflurane group (*n* = 8), the isoflurane group (*n* = 9), the halothane group (*n* = 8), and the propofol group (*n* = 9). This number of animals was calculated to achieve the statistical increase in the arrhythmogenic dose of epinephrine in the sevoflurane group with a *P* value of 0.05 and a power of 90% in our preliminary test. After preparation of brain death and washing out sevoflurane, administration of anesthetics was started in each group setting. In the control group, any anesthetic agent was not exposed, while, in the sevoflurane, isoflurane, and halothane group, animals were inhaled 0.8% of sevoflurane, 0.5% of isoflurane, and 0.3% of halothane, respectively, which is equivalent to 30% of ED_50_ of alveolar concentration of each volatile anesthetic that was determined based on previous studies [8, 9]. In the propofol group, animals were continuously infused low dose of propofol (195 *μ*g*·*kg^−1^ *·*min^−1^), which is 30% of ED_50_ of propofol (650 *μ*g*·*kg^−1^ *·*min^−1^) [10]. The anesthetics were maintained for a further 20 minutes to be saturated; then, epinephrine injection started to determine the arrhythmogenic dose of epinephrine.

### 2.1. Determination of Arrhythmogenic Dose of Epinephrine

The arrhythmogenic dose of epinephrine was defined as the dose that produced three or more premature ventricular contractions within 15 s of injection. Epinephrine was injected at logarithmically spaced doses (0.5, 0.71, 1.0, 1.41, 2.0, 2.83, 4.0, 5.67, 8.0, 11.4 *μ*g*·*kg^−1^, etc.) following initial dose of 4.0 *μ*g*·*kg^−1^ and the concentration of epinephrine was adjusted to inject the epinephrine volume of 0.2 mL. According to our previous report, the 4.0 *μ*g*·*kg^−1^ dose of epinephrine served as an indicator for the direction of subsequent doses of epinephrine to establish the arrhythmogenic dose, that is, lower or higher dose of epinephrine [11]. This method reduces the number of epinephrine injections necessary to determine the arrhythmogenic dose. A period of 10–30 min was allowed between injections until the arterial blood pressure and heart rate became stable. When the arrhythmias (3 or more premature ventricular contractions within 15 s) occurred, 2.0 mL arterial blood samples were collected for the measurement of the plasma concentration of epinephrine. The blood samples were put into precooled plastic tubes containing 20 *μ*L of 200 mM EDTA-2Na and 200 mM Na_2_S_2_O_5_, which were centrifuged at 3600 g for 10 min at 4°C to separate the plasma. For analysis of epinephrine, 0.5 mL plasma was acidified by the addition of 0.25 mL of 2.5% perchloric acid to precipitate protein. The samples were stored at −80°C for no longer than 7 days, until analysis. The plasma concentration of adrenaline was determined in a fully automated high-performance liquid chromatography-fluorimetric system (HLC-8030 Catecholamine Analyzer; Tosoh, Tokyo, Japan), by a diphenylethylenediamine condensation method. This assay method has a limit of sensitivity of 10 pg*·*mL^−1^ for epinephrine and the inter- and intra-assay variation were less than 3%.

### 2.2. Western Blot Analysis

Separate experiments (*n* = 4 per each group) served to determine rat left ventricular levels of connexin 43 phosphorylation 30 min after administration of each anesthetic (one hour after induction of brain death) (Figure 1). After the completion of perfusion, the left ventricle was quickly frozen in liquid nitrogen and stored at −80°C until use. Tissue was homogenized with a Polytron homogenizer (Brinkmann Instruments, Inc., Westbury, NY) in tissue protein extraction reagent (Thermo Fisher Scientific Inc., Waltham, US) with protease and phosphatase inhibitor cocktail (Thermo Fisher Scientific Inc.). The homogenates were centrifuged at 10,000 g for 15 min at 4°C to remove cellular debris and isolate total protein. Protein concentration was determined by the Bradford method (Bio-Rad Laboratories, Hercules, CA). Equivalent amounts (15 *μ*g) of protein samples were mixed with sample buffer. Samples were run on SDS-PAGE (10% polyacrylamide gel; Bio-Rad) and electrotransferred to a polyvinylidene difluoride filter membrane (Hybond P; GE Healthcare UK Ltd., Amersham Place, Little Chalfont, Buckingham-shire HP7 9NA, UK). The following immunodetection steps were performed using SNAP i.d. Protein Detection System (Millipore, Billerica, MA) according to the manuscript script. To reduce nonspecific binding, blocking solution (TOYOBO Co., Ltd., Osaka, Japan) was added to the blot and immediately vacuumed. The blot was then incubated for 10 min at room temperature with primary antibody recognizing connexin 43. We used a mouse monoclonal anti-connexin 43 antibody (1 : 3000; Invitrogen Corporation, Camarillo, CA). After washing the blot with TBS containing 0.05% Tween 20, the blot was incubated for 10 min at room temperature with a suitable secondary antibody conjugated to Horseradish Peroxidase (1 : 6000; GE Healthcare UK Ltd.). To determine the amount of *β*-actin, we used a rabbit polyclonal anti-*β*-actin antibody HRP-conjugated (1 : 6000; Medical & Biological Laboratories Co., Ltd., Nagoya, Japan). The blot was washed with TBS containing 0.05% Tween 20 and subsequently visualized with an enhanced chemiluminescence detection reagent (Luminata Forte Western HRP Substrates; Millipore). Quantitative analysis of the band densities was performed using Image Lab software (Bio-Rad).

### 2.3. Statistical Analysis

All values in the text and figures are presented as mean ± SD. For hemodynamic data, repeated measures analysis of variance was used to evaluate differences over time between groups. For all other data, one-way analysis of variance was used. The multiple comparisons between groups were assessed by Tukey test. All analyses were conducted with SPSS version 14.0. *P* < 0.05 was considered statistically significant.

## 3. Results and Discussion

**Figure 2:**
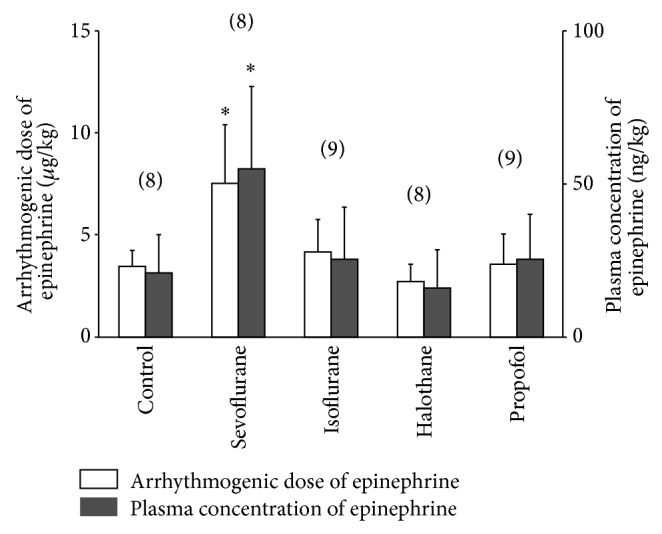
Arrhythmogenic dose (open columns) and plasma concentration (solid columns) of epinephrine in the presence of subanesthetic dose of sevoflurane, isoflurane, halothane, and propofol and no anesthetics (control). The values are expressed as mean ± SD and the number of observations is shown in parentheses. ^*^ *P* < 0.05, compared with the control group.

Figure 2 shows the arrhythmogenic dose and plasma concentration of epinephrine in the presence of subanesthetic dose of sevoflurane, isoflurane, halothane, and propofol and no anesthetics (control). The arrhythmogenic dose of epinephrine in the control group was 3.45 ± 0.79 (mean ± SD) *μ*g*·*kg^−1^ and sevoflurane significantly increased the arrhythmogenic dose and the plasma concentration of epinephrine compared with the other groups (*P* < 0.01). On the other hand, there were no significant differences in arrhythmogenic doses and plasma concentrations of epinephrine among the control, isoflurane, halothane, and propofol groups.

The most important demerit of halothane is to potentiate arrhythmogenic effect of epinephrine and combined application of halothane and epinephrine is contraindication in clinical situations [2]. According to our previous experiment [12], arrhythmogenic dose of epinephrine during halothane anesthesia in naïve rats was 2.35 ± 0.72 *μ*g*·*kg^−1^. On the other hand, the present study showed that the mean arrhythmogenic dose of epinephrine is 3.45 *μ*g*·*kg^−1^ in the control group (no anesthetic after brain death) (Figure 2). This value is a little bit higher than that during halothane in naïve rats, suggesting that epinephrine may produce the arrhythmias easily after brain death. In clinical situations, it is common to administer catecholamines to maintain adequate perfusion for viable organs for transplantation in management of the heart beating brain dead donor, so the present data may reconfirm that catecholamines may significantly increase potential risk for the genesis of arrhythmias after brain death.

In comparison with halothane, isoflurane and sevoflurane have smaller potentiation of epinephrine-induced arrhythmias in humans as well as naive animals [2, 13, 14]. However, the present data showed that the arrhythmogenic doses of epinephrine between halothane and isoflurane were comparable, while the dose in sevoflurane was significantly higher (Figure 2). Thus, the myocardial sensitizing potency of each volatile anesthetic is different between normal and brain dead conditions. The mechanism involved in this discrepancy between normal and brain dead conditions is obscure. It is demonstrated that the catecholamine storm following brain death causes desensitization of myocardial *β*-adrenergic receptors [15]. Thus, it may be possible that the arrhythmogenic potency of epinephrine is also significantly attenuated after brain death. On the other hand, myocardial injury due to brain death may enhance irritability of the heart itself and the present data suggest that sevoflurane is most effective in suppressing the arrhythmias in the heart damaged after brain death.

**Figure 3:**
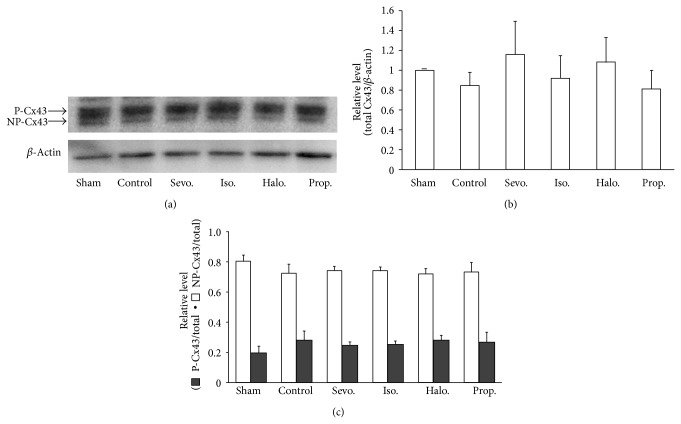
Western blotting analysis of connexin 43 in heart in the presence of subanesthetic dose of sevoflurane, isoflurane, halothane, and propofol and no anesthetics (control) one hour after brain death and the sham group (without brain death). Representative Western blots showing phosphorylated connexin 43 and nonphosphorylated connexin 43 (a). The total connexin 43 calculated as the sum of phosphorylated and nonphosphorylated connexin 43. The total connexin level/*β*-actin level obtained in the sham group (without brain death) is normalized as 1.0 (b). The ratio of phosphorylated connexin 43/nonphosphorylated connexin 43 (c). The values are expressed as mean ± SD and the number of observations is 4 in each group.

In order to explore mechanism involved in the difference of antiarrhythmic effect of sevoflurane and other anesthetics after brain death, we examined the involvement of connexin with Western blot analysis. The result of Western blotting is shown in Figures 3(a)–3(c). The slower migrating major bands represent phosphorylated connexin 43, whereas the faster migrating band is nonphosphorylated connexin 43 (Figure 3(a)) [16]. We calculated the total connexin 43 as the sum of phosphorylated and nonphosphorylated connexin 43 (Figure 3(b)) and showed the ratio of phosphorylated connexin 43/nonphosphorylated connexin 43 (Figure 3(c)). The total connexin 43 prepared one hour after brain death did not change compared with the sham group (without brain death) (Figure 3(b)) and the ratio of phosphorylated connexin 43/total connexin 43 was also similar among the study groups (Figure 3(c)). Thus, total and phosphorylation of connexin are not involved in the antiarrhythmic property of sevoflurane after brain death.

Hemodynamic variables including arterial blood pressure and heart rate are known to be important factors in modulating the genesis of adrenaline-induced arrhythmias [2]. Hemodynamic data before brain death, 30 minutes after brain death or before administration of epinephrine were not different among the study groups (Table 1), suggesting that treatment of any anesthetic at subanesthetic dose did not exert significant effect on hemodynamic data. Furthermore, hemodynamic data at the onset of arrhythmias in the sevoflurane group were not significantly different from those in other groups, despite a larger plasma epinephrine concentration (Table 1 and Figure 2). These results might be due to desensitization of myocardial *β*-adrenergic receptors following brain death [15], which attenuated the hemodynamic changes to the larger plasma epinephrine concentration.

The present data that prophylactic administration of sevoflurane at subanesthetic concentration exerts significant antiarrhythmic effect may have clinical relevance. Control of the lethal arrhythmias is an important issue for management for the organ retrieval and administration of antiarrhythmic drugs may be standard. However, prophylactic efficacy of these drugs after brain death has not been well investigated. Thus, application of subanesthetic sevoflurane may be justified because of this effect without significant further deterioration of hemodynamic situations after brain death. The present data suggest that administration of sevoflurane may be added to an option to control arrhythmias in management of brain dead donor until transplantation including the organ retrieval, when the donor has a risk of genesis of serious arrhythmias. Furthermore, this option would be clinically helpful, especially when the arrhythmias cannot be well controlled by conventional treatment using antiarrhythmic drugs.

We have to discuss potential limitations in our study. First, we anesthetized animals with sevoflurane until induction of brain death. The present study focused on the antiarrhythmic effect of anesthetics following brain death, so we chose sevoflurane to minimize the effect of the anesthetic on our data because of its low solubility and rapid elimination via the lungs and little myocardial sensitizing effect on epinephrine [14]. However, we did not entirely exclude the possibility that the sevoflurane affected the antiarrhythmic property of another anesthetic we tested. Second, the brain death model we used was more controlled than the actual brain death which may occur as a result of various clinical conditions. Thus, the applicability of the present results to the clinical conditions should be prudent owing to the animal model used here.

## 4. Conclusions

Prophylactic administration of subanesthetic dose of sevoflurane, but not halothane, isoflurane, or propofol, is effective to prevent epinephrine-induced arrhythmias after brain death.

## Figures and Tables

**Table 1a:** (a) Mean arterial pressure

Anesthetic agents	*n*	Before brain death	30 min after brain death	Before epinephrine	At the arrhythmias
Control	8	62 ± 19	61 ± 10	60 ± 10	185 ± 15
Sevoflurane	8	81 ± 18	52 ± 3	50 ± 2	175 ± 13
Isoflurane	9	74 ± 17	59 ± 15	51 ± 8	166 ± 24
Halothane	8	79 ± 20	59 ± 13	60 ± 15	177 ± 8
Propofol	9	83 ± 15	57 ± 11	63 ± 15	172 ± 18

**Table 1b:** (b) Heart rate

Anesthetic agents	*n*	Before epinephrine	At the arrhythmias	Before epinephrine	At the arrhythmias
Control	8	281 ± 19	278 ± 25	278 ± 26	355 ± 28
Sevoflurane	8	282 ± 31	282 ± 36	240 ± 19	353 ± 34
Isoflurane	9	279 ± 24	292 ± 23	263 ± 37	358 ± 17
Halothane	8	272 ± 26	264 ± 37	256 ± 26	348 ± 37
Propofol	9	272 ± 23	268 ± 26	252 ± 36	327 ± 32

## References

[1] McKeown D. W., Bonser R. S., Kellum J. A. (2012). Management of the heartbeating brain-dead organ donor. *British Journal of Anaesthesia*.

[2] Atlee J. L., Bosnjak Z. J. (1990). Mechanisms for cardiac dysrhythmias during anesthesia. *Anesthesiology*.

[3] Hirata N., Kanaya N., Kamada N., Kimura S., Namiki A. (2009). Differential effects of propofol and sevoflurane on ischemia-induced ventricular arrhythmias and phosphorylated connexin 43 protein in rats. *Anesthesiology*.

[4] Bugge J. F. (2009). Brain death and its implications for management of the potential organ donor. *Acta Anaesthesiologica Scandinavica*.

[5] Takamatsu T. (2008). Arrhythmogenic substrates in myocardial infarct. *Pathology International*.

[6] Iwasaki M., Hayashi Y., Yamanaka H., Kamibayashi T., Mashimo T. (2011). Nicorandil preserves myocardial function following brain death in rats by mitochondrial adenosine triphosphate-sensitive potassium channel-dependent mechanism. *European Journal of Cardio-thoracic Surgery*.

[7] Pratschke J., Wilhelm M. J., Kusaka M., Laskowski I., Tilney N. L. (2000). A model of gradual onset brain death for transplant-associated studies in rats. *Transplantation*.

[8] Mazze R. I., Rice S. A., Baden J. M. (1985). Halothane, isoflurane, and enflurane MAC in pregnant and nonpregnant female and male mice and rats. *Anesthesiology*.

[9] Taheri S., Halsey M. J., Liu J., Eger E. I., Koblin D. D., Laster M. J. (1991). What solvent best represents the site of action of inhaled anesthetics in humans, rats, and dogs?. *Anesthesia and Analgesia*.

[10] Carmichael F. J., Crawford M. W., Khayyam N., Saldivia V. (1993). Effect of propofol infusion on splanchnic hemodynamics and liver oxygen consumption in the rat: a dose-response study. *Anesthesiology*.

[11] Takada K., Sumikawa K., Kamibayashi T. (1993). Comparative efficacy of antiarrhythmic agents in preventing halothane-epinephrine arrhythmias in rats. *Anesthesiology*.

[12] Iwasaki M., Hayashi Y., Kamibayashi T., Yamatodani A., Mashimo T. (2008). The antiarrhythmic effect of centrally administered rilmenidine involves muscarinic receptors, protein kinase C and mitochondrial signalling pathways. *The British Journal of Pharmacology*.

[13] Johnston R. R., Eger E. I., Wilson C. (1976). A comparative interaction of epinephrine with enflurane, isoflurane, and halothane in man. *Anesthesia and Analgesia*.

[14] Hayashi Y., Sumikawa K., Tashiro C., Yamatodani A., Yoshiya I. (1988). Arrhythmogenic threshold of epinephrine during sevoflurane, enflurane, and isoflurane anesthesia in dogs. *Anesthesiology*.

[15] D'Amico T. A., Meyers C. H., Koutlas T. C. (1995). Desensitization of myocardial *β*-adrenergic receptors and deterioration of left ventricular function after brain death. *The Journal of Thoracic and Cardiovascular Surgery*.

[16] Ren P., Mehta P. P., Ruch R. J. (1998). Inhibition of gap junctional intercellular communication by tumor promoters in connexin43 and connexin32-expressing liver cells: cell specificity and role of protein kinase C. *Carcinogenesis*.

